# The relationships between gender, psychopathic traits and self-reported delinquency: a comparison between a general population sample and a high-risk sample for juvenile delinquency

**DOI:** 10.1186/s13034-017-0202-3

**Published:** 2017-12-19

**Authors:** L. E. W. Leenarts, C. Dölitzsch, T. Pérez, K. Schmeck, J. M. Fegert, M. Schmid

**Affiliations:** 1 Forschungsabteilung, Kinder- und Jugendpsychiatrische Klinik, Universitäre Psychiatrische Kliniken (UPK), Schanzenstrasse 13, 4056 Basel, Switzerland; 2 Klinik für Kinder- und Jugendpsychiatrie/Psychotherapie, Universitätsklinikum Ulm, Steinhövelstrasse 5, 89075 Ulm, Germany

## Abstract

**Background:**

Studies have shown that youths with high psychopathic traits have an earlier onset of delinquent behavior, have higher levels of delinquent behavior, and show higher rates of recidivism than youths with low psychopathic traits. Furthermore, psychopathic traits have received much attention as a robust indicator for delinquent and aggressive behavior in both boys and girls. However, there is a notable lack of research on gender differences in the relationship between psychopathic traits and delinquent behavior. In addition, most of the studies on psychopathic traits and delinquent behavior were conducted in high-risk samples. Therefore, the first objective of the current study was to investigate the relationship between psychopathic traits and specific forms of self-reported delinquency in a high-risk sample for juvenile delinquency as well as in a general population sample. The second objective was to examine the influence of gender on this relationship. Finally, we investigated whether the moderating effect of gender was comparable in the high-risk sample for juvenile delinquency and the general population sample.

**Methods:**

Participants were 1220 adolescents of the German-speaking part of Switzerland (N = 351 high-risk sample, N = 869 general population sample) who were between 13 and 21 years of age. The Youth Psychopathic traits Inventory (YPI) was used to assess psychopathic traits. To assess the lifetime prevalence of the adolescents’ delinquent behavior, 15 items derived from a self-report delinquency instrument were used. Logistic regression analyses were used to examine the relationship between gender, psychopathic traits and self-reported delinquency across both samples.

**Results:**

Our results demonstrated that psychopathic traits are related to non-violent and violent offenses. We found no moderating effect of gender and therefore we could not detect differences in the moderating effect of gender between the samples. However, there was a moderating effect of sample for the relationship between the callous and unemotional YPI scale and non-violent offenses. In addition, the regression weights of gender and sample were, for non-violent offenses, reduced to non-significance when adding the interaction terms.

**Conclusions:**

Psychopathic traits were found to be present in a wide range of youths (i.e., high-risk as well as general population sample, young children as well as adolescents, boys as well as girls) and were related to delinquent behavior. The influence of age and YPI scales on self-reported delinquency was more robust than the influence of gender and sample. Therefore, screening for psychopathic traits among young children with psychosocial adjustment problems seems relevant for developing effective intervention strategies.

## Background

In recent years there has been an increasing interest in the manifestation and assessment of psychopathic traits in children and adolescents [1–3]. Studies have shown that youths with high psychopathic traits have an earlier onset of delinquent behavior, have higher levels of delinquent behavior, and show higher rates of recidivism than youths with low psychopathic traits [4, 5]. Furthermore, in conduct-problem youths, it has been found that the presence of psychopathic traits was related to a more severe pattern of antisocial behavior than when these traits were not present [4]. For example, as found in a study by Lindberg et al. [6] adolescent male homicide offenders scoring high on psychopathic traits, more frequently used excessive violence in their crimes. These findings are in agreement with many previous reports showing that juvenile offenders with psychopathic traits form a special subgroup [4]. Recognizing their characteristics would facilitate effective intervention efforts. However, up till now the vast majority of research on psychopathic traits and delinquent behavior has focused on high-risk samples for juvenile delinquency [7]. While, when defining effective intervention efforts, it is important to test whether the predictive value of psychopathic traits on delinquent behavior is confined only to the most antisocial youths or whether the relationship between psychopathic traits and delinquent characteristics is similar for juvenile justice and non-juvenile justice youths [7].

The few studies focusing on psychopathic traits in non-juvenile justice youths demonstrate that psychopathic traits are highly associated with delinquent behavior. For example, Oshukova et al. [8] found that in a community sample, in both boys and girls, psychopathic traits were highly correlated with rule-breaking and aggressive behavior. In addition, the correlation between psychopathic traits and rule-breaking behavior was significantly higher in boys than in girls. The relationship between psychopathic traits and delinquency among adolescents in residential care (i.e., residing non-juvenile justice youths) is unknown, as studies in these settings are scarce. However, a Dutch study on adolescents in residential care [9] identified that youths scoring high on all three YPI scales scored higher on externalizing problem behavior compared to youths with average scores on the YPI scales. In addition, Schmid et al. [10] reported that youths with psychopathic traits are two to three times more likely to drop out of residential care (i.e., unscheduled termination of measurement by the institution, juvenile or other involved people; e.g., expulsion from the institution because of aggressive behavior towards professionals or other juveniles in the institution, little cooperation from the family of the juvenile, no educational opportunities).

There is a controversial discussion about differences between boys and girls in the manifestation of psychopathic traits and its relation to delinquent behavior. Psychopathic traits are believed to exist in both boys and girls [11, 12]. In addition, in both boys and girls elevated psychopathic traits are related to a higher likelihood of delinquent behavior [4]. However, a number of studies have demonstrated that the relationship between psychopathic traits and delinquent behavior is different for boys and girls (e.g., [4, 7]). For example, the results of a meta-analysis by Asscher et al. [4] showed that the effect size of psychopathy on delinquent behavior was larger in adolescent female samples than in adolescent male samples. An explanation for this finding may be that the relatively small group of girls showing psychopathic traits is a highly disturbed and burdened group, showing high levels of delinquent behavior. Whereas Penney and Moretti [13] found that the relationship, in a high-risk sample, between psychopathic features, aggression and antisocial behavior was equivalent for boys and girls. Generally speaking, psychopathic traits have received much attention as a robust indicator for delinquent and aggressive behavior in both boys and girls. However, there is a notable lack of research on gender differences in the relationship between psychopathic traits and delinquent behavior [13]. In addition, as previously mentioned, most of the studies on psychopathic traits and delinquent behavior were conducted in high-risk samples.

Consequently, the first objective of the current study was to investigate the relationship between psychopathic traits and specific forms of self-reported delinquency in a high-risk sample for juvenile delinquency as well as in a general population sample. As different combinations of elevated scores on psychopathic traits may lead to different types of juvenile delinquency [9], with for example a higher score on all three YPI scales predicting the probability for having committed violent offenses and a higher score on only one scale of the YPI predicting the probability for having committed non-violent offenses, we categorized the self-reported delinquency in two types of offenses (i.e., violent offenses and non-violent offenses).^1^ Furthermore, given the controversial discussion about the role of gender in the relationship between psychopathic traits and specific forms of self-reported delinquency; the second objective was to examine the influence of gender on this relationship. Finally, we investigated whether the moderating effect of gender was comparable in the high-risk sample for juvenile delinquency and the general population sample. Gaining greater understanding of associations between psychopathic traits and delinquent behavior in a high-risk sample for juvenile delinquency as well as in a general population sample is essential for developing effective intervention strategies.

## Methods

### Procedure

The current study was part of the larger *Swiss study for clarification and goal*-*attainment in youth welfare and juvenile justice institutions*, involving the standardized monitoring and evaluation of mental health problems of youths in welfare and juvenile justice institutions in Switzerland [14]. At the same time, the Youth Psychopathic traits Inventory (YPI) and the self-reported delinquency questionnaire were applied to a school sample [15], to obtain data from the general population for purposes of comparison.

The high-risk sample for juvenile delinquency was recruited from 38 welfare and juvenile justice institutions from the German speaking part of Switzerland. Adolescents between 13 and 21 years of age who were admitted to one of the 38 facilities between 2007 and 2011 were asked to participate; with the exception of those who had a placement shorter than 1 month and those who, due to language problems, were not able to complete the assessment tools. Adolescents and their primary caregivers were individually approached by trained staff of the institution who explained the aims and nature of the study. Following Swiss legislation, active informed consent was collected and, if the adolescent was younger than age 18, parental/primary caregiver informed consent was obtained as well. The study was reviewed by the Ethics Review Committees of Basel, Lausanne (Switzerland) and Ulm (Germany). It is important to note that in Switzerland, youths can be placed in welfare and juvenile justice institutions because of: delinquent behavior (*criminal law measure*), youth welfare reasons (*civil law measure,* e.g., maltreatment, parental psychopathology, prostitution and drug abuse) or *other reasons* (e.g., their own or parents’ choice). These three groups currently reside in the same facilities. An analysis by Dölitzsch et al. [16] showed that youths who are placed in youth welfare and juvenile justice institutions because of youth welfare or other reasons, have a high-risk of delinquent behavior: 83.4% reported to have committed at least one offense.

The general population sample was recruited from 18 public schools in the German-speaking part of Switzerland. Schools were selected to cover all curricula and to cover urban as well as rural areas. Youths were included in the study if they were between 13 and 21 years of age and were able to complete the German assessment tools. Assessment took place during a 1-h class. Active informed consent was collected and for minors, parental/primary caregiver informed consent was collected. Participants had a chance to get free movie tickets. The study was reviewed by the Ethics Review Committee of Basel.

### Participants

For the current study, data from 1220 adolescents of the German-speaking part of Switzerland (N = 351 high-risk sample, N = 869 general population sample) who were between 13 and 21 years of age and completed both the YPI [17] and a self-reported delinquency questionnaire [18] were analyzed. Adolescents’ ages, from the high-risk sample, ranged from 13 to 21 years (mean = 16.2, SD = 1.8). Among the 242 (68.9%) boys and 109 (31.1%) girls, 26.6% were placed in the facility under a *criminal law measure*, 55.0% under a *civil law measure* and 18.4% because of *other reasons*. Most adolescents (79.5%) were born in Switzerland and 20.5% was born in other countries. More than one third of the mothers (37.7%) and one fifth (20.2%) of the fathers of youths in the high-risk sample had only finished primary or secondary school. The adolescents’ ages, from the general population sample, ranged from 13 to 21 years (mean = 17.3, SD = 1.3). Among the 497 (57.2%) boys and 372 (42.8%) girls, 86.7% was born in Switzerland and 13.3% was born in other countries. One fourth of the mothers (25%) and 15.3% of the fathers of youths in the general population sample had only finished primary or secondary school.

### Assessment

#### Demographics

Background information (i.e., age, gender and country of birth) for the high-risk sample was extracted by local staff from personal records. Youths from the general population sample answered questions about their personal background in a questionnaire.

#### YPI

The German [Schmeck, Hinrichs & Fegert, 2005, unpublished questionnaire] version of the YPI [17] was used to assess psychopathic traits. The YPI is a self-report questionnaire which consists of 50 items that combine into 10 scales. These scales map onto three domains: grandiose-manipulative (including the subscales dishonest charm, grandiosity, lying and manipulation), callous and unemotional (including the subscales callousness, unemotionality and remorselessness), and impulsive-irresponsible (including the subscales impulsiveness, thrill-seeking and irresponsibility). The respondent rates the questions on a Likert-type four-point rating scale ranging from 1 = does not apply at all to 4 = applies very well. Earlier research on this questionnaire in juvenile justice and non-juvenile justice samples displayed satisfactory psychometric properties [15, 17]. In the current study, Cronbach’s alpha coefficients of the scales ranged from 0.82 to 0.90.

#### Self-reported delinquency

To assess the lifetime prevalence of the adolescents’ delinquent behavior, 15 items derived from a validated instrument [18] were used. The items assess three forms of delinquent behavior, namely: vandalism (3 items), property offenses (8 items) and violent offenses (4 items). Vandalism expresses damage to or the destruction of public or private property, caused by a person who is not its owner. Property offenses refers to the taking of property, and does not involve (threat of) force against a victim or damage to or destruction of the property. Violent offenses refers to crimes in which an offender uses or threatens force upon a victim. This entails both crimes in which the violent act is the objective as well as crimes in which violence is the means to an end. Adolescents were asked anonymously, if they had ever committed the designated delinquent behavior, how old they were when they first committed the behavior and how often they had committed the behavior. For the analyses, the three forms of self-reported delinquency were categorized into two variables: violent offenses versus non-violent offenses (i.e., vandalism and property offenses).

### Statistics

First, we generated descriptive statistics (using Statistical Package for Social Science, SPSS, 21) for the study variables and compared YPI scores, and self-reported delinquency across the two samples via t-test and Chi square analyses.

Next, we conducted logistic regression analyses, for each YPI scale separately, that regressed violent offenses and non-violent offenses on age, YPI scale, gender and sample. In the second block all the two-way interactions were included in the analyses (excluding interactions with age). To test for the potential moderating effect of gender, we checked whether the interaction terms contributed significantly to the regression equation. In the third and final block the three-way interaction between gender, sample and YPI scale was included, to investigate whether the moderating effect of gender was comparable in the high-risk sample and the general population sample.

## Results

### Comparisons across samples

YPI means were compared across the high-risk sample and the general population sample. Youths from the high-risk sample scored significantly higher than youths from the general population sample on all the YPI scales: grandiose-manipulative [10.58 versus 9.38; *t*(587) = 7.06, *p* < 0.001], callous and unemotional [11.01 versus 9.84; *t*(1218) = 7.77, *p* < 0.001], and impulsive-irresponsible [12.92 versus 11.36; *t*(577) = 9.33, *p* < 0.001]. Considering self-reported delinquency; youths from the high-risk sample were more likely than youths from the general population sample to report non-violent offenses [84.3% versus 61.4%; χ^2^(1) = 60.18, *p* < 0.001], and violent offenses [60.1% versus 26.2%; χ^2^(1) = 124.56, *p* < 0.001].

### Logistic regression non-violent offenses

Table 1 presents the models predicting non-violent offenses. First, we considered the YPI grandiose-manipulative scale for non-violent offenses (Table 1, Model 1); the first block significantly predicted non-violent offenses [χ^2^(4) = 177.17, *p* < 0.001; Nagelkerke R^2^ = 0.19]. A significant main effect emerged for age, the YPI grandiose-manipulative scale, gender and sample. The second block revealed no improvement in explained variance compared to the first block [χ^2^(3) = 3.13, *p* = 0.372; Nagelkerke R^2^ = 0.19]. The contributions of age and the YPI grandiose-manipulative scale remained essentially unchanged, while the main effects of gender and sample were reduced to non-significance. The two-way interaction terms did not significantly contribute to the regression equation. The third block, which also included the three-way interaction term, yielded similar results as the second block [χ^2^(1) = 1.39, *p* = 0.238; Nagelkerke R^2^ = 0.19]. The only significant contributors to the equation were age and the YPI grandiose-manipulative scale.

**Table 1:** Logistic regression non-violent offenses

	Model 1 (grandiose-manipulative)			Model 2 (callous and unemotional)			Model 3 (impulsive-irresponsible)		
	B	SE B	Exp (B)	B	SE B	Exp (B)	B	SE B	Exp (B)
Block 1									
Age	0.14	0.05	1.15**	0.14	0.05	1.15**	0.14	0.05	1.15**
YPI scale	0.24	0.03	1.28***	0.20	0.03	1.22***	0.42	0.03	1.53***
Gender (boys = 1, girls = 0)	0.43	0.14	1.53**	0.31	0.14	1.36*	0.50	0.14	1.64***
Sample (high-risk = 1, general = 0)	1.16	0.18	3.20***	1.18	0.18	3.27***	0.97	0.19	2.63***
Block 2									
Age	0.14	0.05	1.15**	0.14	0.05	1.15**	0.14	0.05	1.15**
YPI scale	0.26	0.05	1.30***	0.09	0.05	1.10	0.44	0.06	1.55***
Gender	0.97	0.57	2.64	− 0.56	0.66	0.57	0.90	0.78	2.45
Sample	1.01	0.68	2.74	− 0.27	0.84	0.76	1.03	0.92	2.81
YPI × gender	− 0.05	0.06	0.95	0.11	0.07	1.11	− 0.03	0.07	0.97
YPI × sample	0.05	0.07	1.05	0.19	0.09	1.21*	0.01	0.08	1.01
Gender × sample	− 0.54	0.36	0.58	− 0.63	0.36	0.53	− 0.34	0.37	0.71
Block 3									
Age	0.14	0.05	1.15**	0.14	0.05	1.15**	0.14	0.05	1.15**
YPI scale	0.24	0.05	1.27***	0.10	0.06	1.11	0.44	0.06	1.56***
Gender	0.66	0.63	1.93	− 0.43	0.72	0.65	1.01	0.89	2.74
Sample	− 0.15	1.23	0.86	0.19	1.33	1.21	1.34	1.46	3.81
YPI × gender	− 0.01	0.07	0.99	0.09	0.07	1.10	− 0.04	0.08	0.96
YPI × sample	0.19	0.14	1.21	0.14	0.14	1.15	− 0.02	0.13	0.99
Gender × sample	1.16	1.50	3.20	− 1.39	1.76	0.25	− 0.83	1.86	0.44
YPI × gender × sample	− 0.19	0.17	0.82	0.08	0.18	1.08	0.04	0.16	1.05

*B* unstandardized regression coefficient, *SE B* standard error regression coefficient, *Exp (Β)* expected regression coefficient (odds ratio), *YPI* Youth Psychopathic Traits Inventory

* *p* < 0.05; ** *p* < 0.01; *** *p* < 0.001

Next, we considered the YPI callous and unemotional scale for non-violent offenses (Table 1, Model 2); the first block significantly predicted non-violent offenses [χ^2^(4) = 140.25, *p* < 0.001; Nagelkerke R^2^ = 0.15]. Again, a significant main effect emerged for age, the YPI callous and unemotional scale, gender and sample. Adding all the two-way interactions to the model significantly improved model fit [χ^2^(3) = 9.18, *p* = 0.027; Nagelkerke R^2^ = 0.16]. Regarding the main effects, only the main effect of age remained significant. In addition, the two-way interaction term sample × YPI callous and unemotional contributed significantly to the regression equation. Meaning that having a higher score on the YPI callous and unemotional scale increased the probability for having committed non-violent offenses for youths from the high-risk sample and not for youths from the general population sample. Adding the three-way interaction did not significantly improve model fit [χ^2^(1) = 0.20, *p* = 0.658; Nagelkerke R^2^ = 0.16]. Age was the only significant contributor to this regression equation.

Finally, we considered the YPI impulsive-irresponsible scale for non-violent offenses (Table 1, Model 3). The first block significantly predicted non-violent offenses [χ^2^(4) = 299.81, *p* < 0.001; Nagelkerke R^2^ = 0.30]. Significant main effects emerged for age, the YPI impulsive-irresponsible scale, gender and sample. The second block revealed no improvement in explained variance compared to the first block [χ^2^(3) = 1.12, *p* = 0.772; Nagelkerke R^2^ = 0.31]. The contributions of age and the YPI impulsive-irresponsible scale remained essentially unchanged, while the other main effects were reduced to non-significance. None of two-way interactions contributed substantially to the regression equation. Adding the three-way interaction did not improve model fit [χ^2^(1) = 0.07, *p* = 0.789; Nagelkerke R^2^ = 0.31]. Only age and the YPI impulsive-irresponsible scale contributed significantly to this regression equation.

### Logistic regression violent offenses

Considering the YPI grandiose-manipulative scale for violent offenses (Table 2, Model 1); the first block significantly predicted violent offenses [χ^2^(4) = 234.16, *p* < 0.001; Nagelkerke R^2^ = 0.24]. A significant main effect emerged for age, the YPI grandiose-manipulative scale, gender and sample. The second block revealed a significant improvement in explained variance compared to the first block [χ^2^(3) = 9.57, *p* = 0.023; Nagelkerke R^2^ = 0.25]. All main effects remained essentially unchanged. In addition, the two-way interaction term gender x sample contributed significantly to the regression equation. Meaning that in the high-risk sample there was no difference between boys and girls in the probability of having committed violent offenses, while in the general population sample boys had a higher probability of having committed violent offenses than girls. In addition, in girls the probability of having committed violent offenses was higher when the girl was from the high-risk sample than when she was from the general population sample. In boys there was no difference between the high-risk sample and the general population sample in the probability of having committed violent offenses. Adding the three-way interaction term did not improve model fit [χ^2^(1) = 0.84, *p* = 0.360; Nagelkerke R^2^ = 0.25]. Only age and the YPI grandiose-manipulative scale contributed significantly to this regression equation.

**Table 2:** Logistic regression violent offenses

	Model 1 (grandiose-manipulative)			Model 2 (callous and unemotional)			Model 3 (impulsive-irresponsible)		
	B	SE B	Exp (B)	B	SE B	Exp (Β)	B	SE B	Exp (B)
Block 1									
Age	0.11	0.05	1.12*	0.13	0.05	1.13**	0.11	0.05	1.12*
YPI scale	0.17	0.03	1.18***	0.24	0.03	1.27***	0.23	0.03	1.26***
Gender (boys = 1, girls = 0)	0.86	0.15	2.37***	0.62	0.15	1.86***	0.96	0.15	2.62***
Sample (high-risk = 1, general = 0)	1.41	0.15	4.11***	1.42	0.15	4.14***	1.29	0.16	3.63***
Block 2									
Age	0.12	0.05	1.13**	0.13	0.05	1.14**	0.12	0.05	1.13**
YPI scale	0.23	0.06	1.26***	0.22	0.06	1.25***	0.30	0.06	1.35***
Gender	1.75	0.61	5.78**	0.56	0.71	1.75	2.34	0.76	10.38**
Sample	2.35	0.58	10.49***	2.13	0.68	8.42**	2.02	0.76	7.54**
YPI × gender	− 0.06	0.06	0.94	0.03	0.07	1.03	− 0.09	0.06	0.91
YPI × sample	− 0.04	0.05	0.96	− 0.02	0.07	0.98	− 0.02	0.06	0.98
Gender × sample	− 0.80	0.31	0.45**	− 0.72	0.32	0.49*	− 0.70	0.31	0.50*
Block 3									
Age	0.12	0.05	1.13**	0.13	0.05	1.14**	0.12	0.05	1.13**
YPI scale	0.19	0.07	1.21**	0.25	0.07	1.29***	0.29	0.07	1.34***
Gender	1.33	0.76	3.77	0.97	0.88	2.64	2.23	0.97	9.26*
Sample	1.57	1.04	4.80	2.91	1.20	18.41*	1.82	1.31	6.15
YPI × gender	− 0.02	0.08	0.98	− 0.01	0.09	0.99	− 0.08	0.08	0.92
YPI × sample	0.05	0.11	1.05	− 0.10	0.12	0.90	0.00	0.10	1.00
Gender × sample	0.30	1.25	1.35	− 1.86	1.48	0.16	− 0.41	1.57	0.67
YPI × gender × sample	− 0.11	0.12	0.89	0.11	0.14	1.12	− 0.02	0.12	0.98

*B* unstandardized regression coefficient, *SE B* standard error regression coefficient, *Exp (Β)* expected regression coefficient (odds ratio), *YPI* Youth Psychopathic Traits Inventory

* *p* < 0.05; ** *p* < 0.01; *** *p* < 0.001

Next, we considered the YPI callous and unemotional scale for violent offenses (Table 1, Model 2); the first block significantly predicted violent offenses [χ^2^(4) = 254.85, *p* < 0.001; Nagelkerke R^2^ = 0.26]. Again, a significant main effect emerged for age, the YPI callous and unemotional scale, gender and sample. The second block revealed no improvement in explained variance compared to the first block [χ^2^(3) = 6.21, *p* = 0.102; Nagelkerke R^2^ = 0.26]. Regarding the main effects, all remained the same, except for gender. Gender no longer contributed significantly to the regression equation. Considering the two-way interactions, as in Model 1 for violent offenses gender × sample contributed significantly to the regression equation. Adding the three-way interaction term did not improve model fit [χ^2^(1) = 0.62, *p* = 0.432; Nagelkerke R^2^ = 0.26]. All main effects remained the same. Neither the two-way interactions, nor the three-way interaction contributed significantly to the regression equation.

Finally, we considered the YPI impulsive-irresponsible scale for violent offenses (Table 1, Model 3). The first block significantly predicted violent offenses [χ^2^(4) = 266.87, *p* < 0.001; Nagelkerke R^2^ = 0.27]. Significant main effects emerged for age, the YPI impulsive-irresponsible scale, gender and sample. The second block revealed a significant improvement in explained variance compared to the first block [χ^2^(3) = 8.61, *p* = 0.035; Nagelkerke R^2^ = 0.28]. A significant main effect emerged for age, the YPI impulsive-irresponsible scale, gender and sample. Considering the two-way interactions, as in Model 1 and 2 for violent offenses gender × sample contributed significantly to the regression analyses. Adding the three-way interaction term did not improve model fit [χ^2^(1) = 0.04, *p* = 0.849; Nagelkerke R^2^ = 0.28]. Only the main effects age, the YPI impulsive-irresponsible scale and gender contributed significantly to this regression equation. Sample no longer contributed significantly to the regression equation. Neither the two-way interactions, nor the three-way interaction contributed significantly to the regression equation.

## Discussion

The purpose of the current study was to examine the relationship between psychopathic traits and self-reported non-violent and violent offenses in a high-risk sample for juvenile delinquency as well as in a general population sample and how gender influences this relationship. We also investigated whether the moderating effect of gender was comparable in the high-risk sample for juvenile delinquency and the general population sample. Consistent with previous research [4, 5], our results demonstrated that psychopathic traits are related to non-violent and violent offenses. We found no moderating effect of gender and therefore we could not detect differences in the moderating effect of gender between the samples. However, there was a moderating effect of sample for the relationship between the callous and unemotional YPI scale and non-violent offenses. Youths from the high-risk sample with a higher score on the YPI callous and unemotional scale had a higher probability for having committed non-violent offenses than youths scoring low on this scale. In youths from the general population sample, this was not the case. Because the three-way interaction YPI callous and unemotional scale × gender × sample was not significant, it can be concluded that the moderating effect of sample was comparable for boys and girls. Considering the moderating effect of sample for the relationship between the callous and unemotional YPI scale and non-violent offenses, surprisingly, youths from the high-risk sample with a higher score on the YPI callous and unemotional scale had a higher probability for having committed *non*-violent offenses than youths scoring low on this scale and this was not the case for violent offenses. An explanation for this finding may be found in the fact that higher scores on all three YPI scales predict the probability for having committed violent offenses [9]. This may indicate that youths with a higher score on only one scale of the YPI can be seen as a less ‘severe’ group of juvenile offenders, committing ‘only’ non-violent offenses, compared to youths with a higher score on all three YPI scales, committing violent offenses.

The regression weights of gender and sample were, for non-violent offenses, reduced to non-significance when adding the interaction terms. Therefore, it can be concluded that the influence of gender and sample on non-violent offenses was less robust than the influence of age and YPI scales. This finding is in line with earlier research reporting that higher levels of psychopathic traits are associated with higher levels of self-reported delinquency [4] and that the involvement in delinquency increases considerably during adolescence [19]. In addition, the level of offenses such as vandalism (i.e., non-violent offenses), peaks at a younger age (i.e., age 14–15), whereas the level of violent offenses peaks at an older age (i.e., age 16–17 [19]). In our sample however, adolescents were asked if they had *ever* committed the designated delinquent behavior. Consequently, the probability of having committed offenses during lifetime increased the older juveniles of this high-risk sample were.

Several limitations should be considered. First, the cross-sectional design of our study may limit the interpretation of our findings. Second, we relied solely on the participants’ self-reported delinquent behavior. As a consequence, under-reporting of delinquent behavior may have occurred. However, analyses have shown that youths from the high-risk sample reported more delinquent behavior than the professional caregivers from their institutions [16]. In addition, psychopathic traits were also measured through self-report only, the socially desirable responding on questions of the YPI may have influenced the scores on the YPI. However, a study by Cauffman et al. [20] demonstrated that self-reported psychopathic traits was a better predictor of self-reported delinquent behavior compared to expert-rated psychopathic traits. Third, the questionnaire for self-reported delinquency included items that assess also mild forms of delinquent behavior (e.g., ‘*Have you ever sprayed graffiti on places were this was illegal?*’, ‘*Have you ever taken something from a supermarket, store or a mall without paying for it?*’) which may explain the relatively high rates of delinquent behavior in both samples. Lastly, we did not include the level of psychopathology in our study. An extensive body of research has documented that a high proportion of especially youths from the high-risk sample meet criteria for psychopathology [22, 23]. Since psychopathic traits have been found to be related to psychopathology (e.g., [8, 9, 21]) and psychopathology has been found to be related to delinquent behavior in youths (e.g., [22–24]), it is reasonable to suggest that the level of psychopathology influences the relationship between psychopathic traits and specific forms of delinquent behavior, and therefore may have influenced our results.

Despite these limitations the current study leads us to formulate a number of recommendations for future research. The YPI displayed satisfactory psychometric properties in juvenile justice and non-juvenile justice samples [15, 17]. However, a study by Colins et al. [25], demonstrated that YPI scores were not able to predict future offending, which may suggest that the YPI should not yet be used for risk assessment purposes. Therefore, future research should investigate the prognostic usefulness of the YPI. Furthermore, currently the YPI uses the same scoring key for boys and for girls, while the identification of personality traits in juvenile justice youths is influenced by gender variations in symptom expression (boys tend to reveal their feelings on self-report scales less readily than girls [26], it may be reasonable to suggest that the current cut-off scores for boys under-detect certain psychopathic traits. Future research should address whether the current scoring key of the YPI adequately detects psychopathic traits in boys as well as in girls. Moreover, YPI norms (e.g., for different age groups, gender and different samples) should be developed to be able to give meaningful interpretations in individual cases. Lastly, it is crucial that further research includes follow-up data to investigate the long term negative outcomes of youths scoring high on psychopathic traits in, for example, contacts with family, relationships, school/work and living situation.

## Conclusion

Overall, the current study contributes to the body of research examining the consequences of psychopathic traits in juveniles. Psychopathic traits are found to be present in a wide range of youths (i.e., high-risk as well as general population sample, young children as well as adolescents, boys as well as girls) and are related to delinquent behavior. This study showed that psychopathic traits are related to non-violent and violent offenses. The influence of age and YPI scales on self-reported delinquency was more robust than the influence of gender and sample. Therefore, based on this study, screening for psychopathic traits among young children with psychosocial adjustment problems seems relevant for developing effective intervention strategies.

## Footnotes

1 The current study focuses on self-reported delinquency, the term delinquency is used as a more general category which is categorized in violent offenses and non-violent offenses.
